# Research on Simulation of Fatigue Crack Growth in LNG Storage Tanks and Prediction of Residual Service Life

**DOI:** 10.3390/ma19102028

**Published:** 2026-05-13

**Authors:** Qingwen Zhang, Xiang Yi, Zhengxin Li, Weixin Zhou, Jingxi Liu

**Affiliations:** 1 China Classification Society, Beijing 100010, China; 2 School of Naval Architecture and Ocean Engineering, Huazhong University of Science and Technology, Wuhan 430074, China

**Keywords:** LNG tanks, high-manganese steel, 3D crack growth, XGBoost, residual life

## Abstract

This study evaluates fatigue crack growth in marine high-manganese steel LNG (Liquefied Natural Gas) storage tanks under cryogenic conditions. A 3D simulation framework using the *M*-integral for stress intensity extraction and the VCTD (Vertical Crack Tip Displacement) criterion for path prediction was developed. Parametric simulations showed that crack propagation is strongly directional, with the surface growth rate exceeding the depthwise rate. Fatigue life decreased with increasing initial crack surface length and maximum load but increased with crack inclination angle. In addition, the Mode I stress intensity factor along the depthwise path converged during propagation and rose sharply when the crack depth approached 90% of the wall thickness. An XGBoost-based dual-target model further achieved accurate prediction of crack depth and residual life.

## 1. Introduction

Driven by increasingly stringent global regulations on carbon emissions, Liquefied Natural Gas (LNG) has emerged as a pivotal cleaner alternative to conventional marine fuels. Consequently, marine LNG fuel storage tanks have become critical components of modern shipboard energy systems. These tanks typically operate under cryogenic conditions while enduring complex dynamic loads and environmental fluctuations during long-term navigation. The fatigue performance of these structures, particularly under cyclic loading, is a decisive factor for structural integrity, service life, and operational reliability. Therefore, robust numerical simulation of fatigue crack growth (FCG) and residual life assessment are essential for mitigating accident risks and ensuring long-term in-service safety. High-manganese austenitic steel is increasingly favored for LNG tank fabrication due to its excellent cryogenic properties. However, defects are inevitably introduced during production and welding, serving as preferential sites for crack initiation. Under continuous service, these cracks may propagate and eventually penetrate the hull, potentially leading to catastrophic gas leakages.

Current FCG (Fatigue Crack Growth) prediction methodologies are primarily rooted in fracture mechanics. While traditional models rely on the Paris Law—establishing an exponential relationship between the Stress Intensity Factor (SIF) and crack growth rate [1]—subsequent studies by Forman et al. [2] and others have incorporated stress ratio *R* and fracture toughness *K*_c_ to enhance applicability. To simulate crack paths, various criteria have been established, including the Maximum Tensile Stress (MTS) criterion [3], the Strain Energy Density (SED) criterion [4,5], and the crack-tip displacement vector criterion based on Crack-Tip Opening Displacement (CTOD) [6].

Among these fracture parameters, Crack-Tip Opening Displacement (CTOD) has garnered significant attention because it directly characterizes the extent of plastic deformation at the crack tip. CTOD is instrumental not only in evaluating material fracture toughness but also in analyzing crack propagation behavior under complex stress states. Kawabata et al. [7] proposed a refined CTOD calculation formula by introducing a correction parameter to account for the strain-hardening exponents of various blunting shapes at the crack front; this approach enables a more accurate determination of fracture toughness for thick sections and non-standard three-point bending specimens. Similarly, Zhen et al. [8] investigated the influence of specimen thickness and pre-crack-tip constraints on fracture toughness, proposing a constraint-modified fracture criterion based on CTOD. To simplify fracture toughness measurements, Silvestre et al. [9] developed a modified three-point bending test scheme that accurately determines CTOD from the crack-mouth opening displacement (CMOD). In the context of low-temperature applications, Gao et al. [10] measured the CTOD of Q690 steel, elucidating the brittle fracture mechanisms of high-strength steel under cryogenic conditions and providing theoretical support for its use in extreme environments. Furthermore, Dover [11] identified a functional relationship between the CTOD range and the fatigue crack growth (FCG) rate, analogous to the Paris law. This was further expanded by Vasco-Olmo et al. [12], who employed digital image correlation (DIC) to decompose the CTOD range into elastic and plastic components. Their findings revealed that the plastic component of the CTOD range and the FCG rate *da*/*dN* exhibit a power-law relationship similar to the classic Paris formula.

With advances in computational technology, numerical methods have significantly accelerated fracture-mechanics-based investigations into fatigue crack growth (FCG). Gesell et al. [13] developed a numerical framework utilizing two-dimensional (2D) finite-element mesh rezoning, where the crack-tip opening displacement (CTOD) range was employed to evaluate the FCG rate and perform low-cycle fatigue analysis on tensile specimens with prefabricated single-edge notches. Similarly, Escalero et al. [14] proposed a simulation approach based on the plastic component of the CTOD range (ΔCTOD), in which crack advancement was modeled via node release coupled with mesh mapping techniques. Further exploring mixed-mode scenarios, Floros et al. [15] and Malíková et al. [16] employed the finite element method to investigate mixed-mode crack propagation and evaluated multiple criteria for predicting crack growth direction; their results indicated that the Vector Crack Tip Displacement (VCTD) criterion offers superior accuracy. Building upon these foundations, Ganesh et al. [17] developed a three-dimensional (3D) crack propagation program based on finite element remeshing. In their framework, CTOD was used to evaluate the crack growth rate, while the VCTD criterion was adopted to determine the propagation angle, with mesh mapping implemented to facilitate the simulation of crack advancement.

In addition to remeshing-based crack-front tracking methods, several advanced numerical strategies have recently been developed for fatigue crack growth simulation. Ammendolea et al. [18] proposed a fatigue crack growth simulation approach based on the moving mesh technique, which improves the treatment of crack advancement without repeatedly reconstructing the entire finite element model. Baktheer et al. [19] extended the phase-field cohesive-zone framework to fatigue crack growth and verified its applicability through two- and three-dimensional benchmark examples. These studies further enrich the available numerical tools for crack growth analysis. In the present work, a remeshing-based three-dimensional crack-front tracking framework is adopted because it can be directly coupled with *M*-integral-based stress intensity factor evaluation, VCTD-based crack path prediction, and local submodel updating for LNG storage tank structures.

Data-driven fatigue life prediction frameworks leverage machine learning to analyze experimental and simulation datasets, quantifying the complex relationships between fatigue life and key influencing factors—such as material properties, structural configurations, crack geometries, and loading conditions. This approach enables precise estimation of structural durability under operational service conditions. Specifically, Ince [20] employed artificial neural networks (ANNs) to model fracture parameters in cementitious materials, demonstrating the robust potential of neural-based approaches in fracture mechanics. Gope et al. [21] constructed an efficient ANN model to forecast the propagation direction of multiple cracks. Furthermore, Wang et al. [22] proposed an ANN-based method to accurately predict fatigue crack paths and residual life under variable amplitude loading by training on stress and positional parameters. Similarly, Baptista et al. [23] utilized the VCTD criterion to simulate crack propagation in modified compact tension (CT) specimens, subsequently using the generated data to develop an ANN model for path prediction. Beyond neural networks, Gan et al. [24] integrated random forests with kernel extreme learning machines to construct a model capable of predicting fatigue life as influenced by mean stress effects. More recently, physics-informed neural network (PINN) approaches have been introduced to incorporate governing physical laws into the learning process, enabling simultaneous parameter identification and residual life prediction for fatigue crack growth, thereby improving model interpretability and prediction accuracy [25]. Building on this trajectory, Liao et al. [26] established a novel PINN framework integrated with configurational force theory to specifically address the nonlinear propagation characteristics of mixed-mode (I–II) fatigue cracks in ductile metallic materials. This methodology achieves integrated and accurate predictions of both propagation paths and residual life, while enabling the inverse identification of critical crack lengths through a genetic algorithm.

Existing studies on crack-tip opening displacement (CTOD) predominantly focus on two-dimensional configurations, while investigations into three-dimensional (3D) cracks remain largely restricted to single-sided through-cracks. In 3D analyses, CTOD is commonly evaluated using the discrete displacement of the first or second node behind the crack tip; however, this approach renders the results highly sensitive to the finite element mesh density [27]. Therefore, developing robust and mesh-independent CTOD evaluation methods for 3D simulations is imperative.

Based on the above review, this study establishes a three-dimensional fatigue crack growth simulation procedure for LNG storage tanks with semi-elliptical surface cracks. In the numerical procedure, the *M*-integral is used to calculate the stress intensity factors along the crack front, the VCTD criterion is used to determine the crack propagation direction, and finite element remeshing is performed to update the crack geometry during growth. The developed procedure is applied to a high-manganese austenitic steel LNG storage tank, and the effects of initial crack surface length, initial crack direction, and maximum load on crack shape evolution, stress intensity factor variation, and fatigue crack growth life are investigated. In addition, the simulation results are used to train XGBoost regression models for residual life prediction and crack depth prediction. The main contribution of this work is therefore the combination of three-dimensional fatigue crack growth simulation, parametric analysis, and data-driven prediction for residual life assessment of LNG storage tanks.

## 2. Experiments on Fatigue Crack Growth Rates of Material

### 2.1. The Mechanical Properties of High Manganese Austenitic Steel

**Figure 1:**
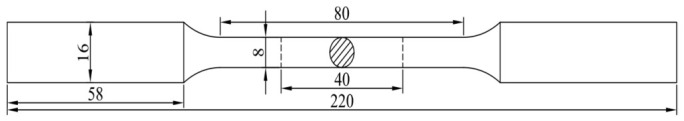
Geometry of the standard R4 cylindrical specimen (all dimensions are in mm).

To obtain the mechanical properties of high-manganese austenitic steel (WuYang Iron and Steel Co., Ltd., Wugang, China), quasi-static tensile tests were conducted in accordance with GB/T 228.1-2010 [28] (equivalent to ISO 6892-1 [29]). To minimize measurement errors associated with specimen size effects, a standard R4 cylindrical specimen was prepared with a gauge length (*L*_0_) of 40 mm and a diameter (*d*_0_) of 8 mm, as illustrated in Figure 1.

**Figure 2:**
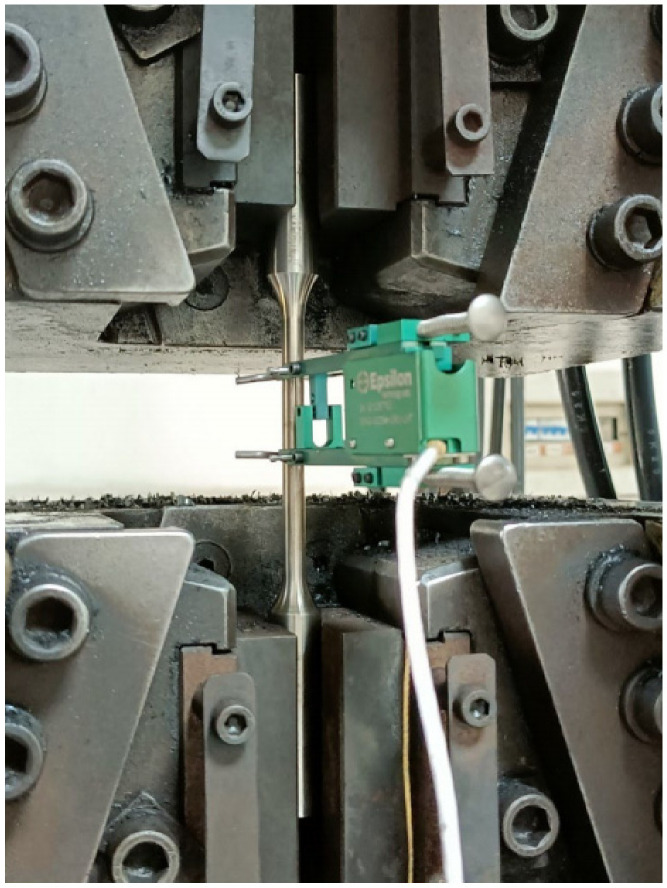
Tensile testing equipment.

**Figure 3:**
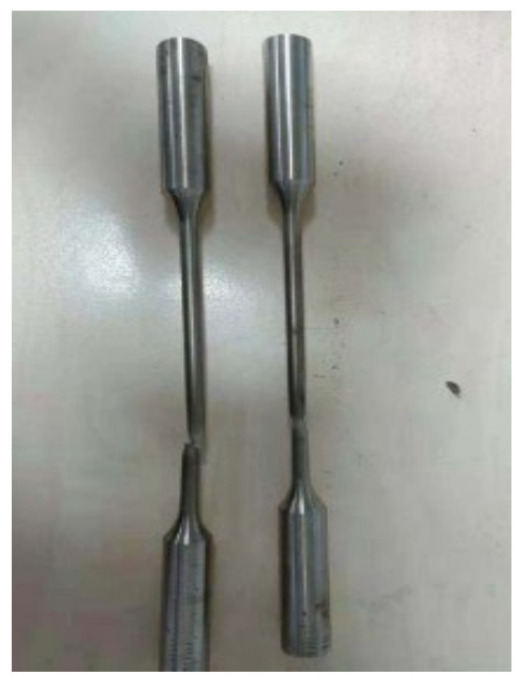
Standard R4 cylindrical specimen after the tensile test.

**Figure 4:**
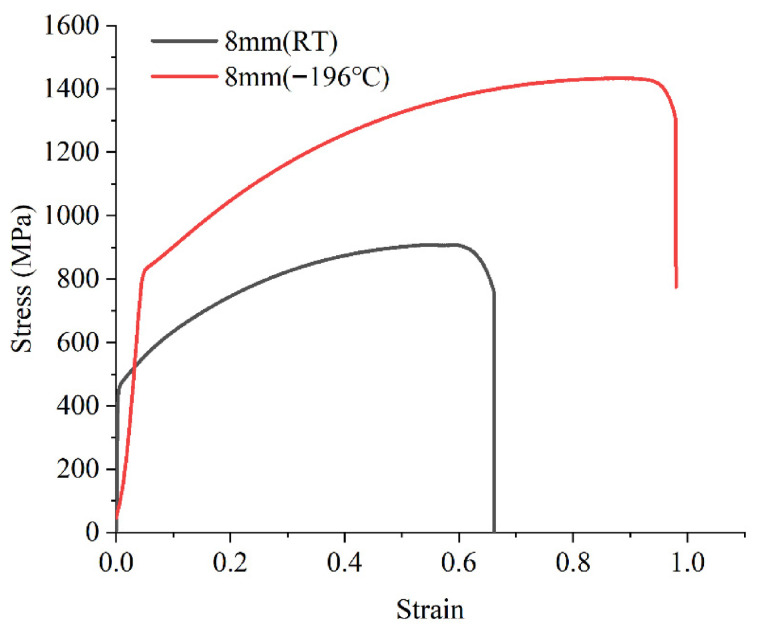
Tensile test results of high manganese austenitic steel at room temperature and −196 °C.

Tensile tests were performed using a 500 kN Shimadzu electronic universal testing machine (Japan), with the experimental setup illustrated in Figure 2. The post-fracture morphology of a standard specimen is shown in Figure 3. Table 1 summarizes the measured mechanical properties at room temperature. The material exhibits an excellent combination of strength and ductility, with a yield strength of 480 MPa and a reduction in area of 60%. The engineering stress–strain curve is depicted in Figure 4.

### 2.2. The Specimens and Crack Growth Experiments

Fatigue crack growth tests were conducted according to the GB/T 6398-2017 standard [30] (equivalent to ISO 12108:2012 [31]). Standard Single Edge Notch Bend (SE(B)) specimens were used with dimensions following the ratio S:W:B = 4:1:0.5, where *S* is the span (100 mm), *W* is the width (25 mm), and *B* is the thickness (12.5 mm). Tests were carried out at both room temperature and cryogenic temperature to characterize the temperature sensitivity of the material.

The crack growth behavior is described by the Paris Law [1], $da/dN={C(∆K)}^{m}$, where *da*/*dN* is the fatigue crack growth rate, Δ*K* is the stress intensity factor range, and *C* and m are material parameters determined from the fatigue crack growth test data. By fitting the measured *da*/*dN*-Δ*K* data, the Paris-law exponent *m* and coefficient *C* adopted in this study were determined as 3.245 and 4.17 × 10^−9^, respectively.

## 3. Static Strength Analysis of LNG Storage Tanks

### 3.1. LNG Storage Tank Geometric Model

**Figure 5:**
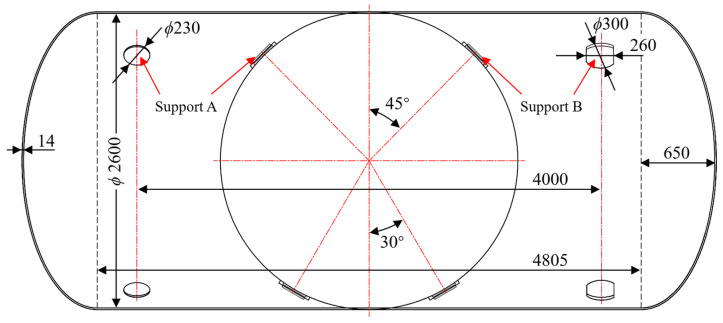
LNG storage tank geometric model and dimensions (all dimensions are in mm).

Taking a 30 m^3^ marine LNG storage tank as a case study, natural gas leakage is primarily attributed to through-thickness cracks originating from the inner cylindrical shell. Since fatigue crack growth is typically confined to localized regions, the tank model can be simplified into three main components: a cylindrical section, ellipsoidal heads, and supporting structures. The simplified geometric model and its corresponding dimensions are illustrated in Figure 5. The tank features a wall thickness of 14 mm, an external diameter of 2600 mm, and a cylindrical section length of 4805 mm, with the ellipsoidal head height measuring 650 mm. The assembly is secured by eight restraints categorized into two distinct support types: Support A, a cylindrical column with a 230 mm diameter; and Support B, a hybrid structure consisting of a 300 mm diameter arc segment and a 260 mm wide straight segment. The longitudinal spacing between Support A and Support B is 4000 mm.

### 3.2. LNG Storage Tank Finite Element Model

**Figure 6:**
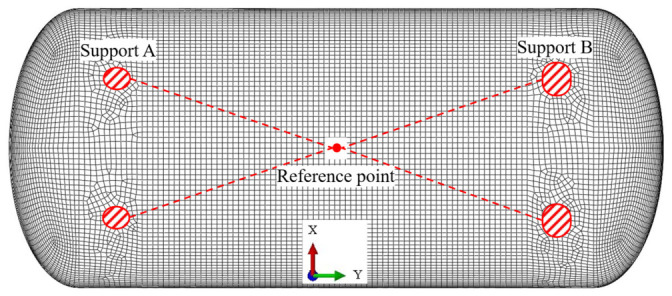
Finite element model of LNG storage tank.

Finite element (FE) modeling and simulations were conducted using the commercial software ABAQUS 2022 (Dassault Systèmes Simulia Corp., Providence, RI, USA). The FE model, established based on the geometry and dimensions of the LNG storage tank, is illustrated in Figure 6. The high-manganese austenitic steel was characterized as a linearly elastic material, with an elastic modulus of 179 GPa and a Poisson’s ratio of 0.3. To facilitate boundary conditions, a reference point (RP) was defined at the geometric center of the tank. Kinematic coupling constraints were applied between the reference point and the nodes on the tank’s outer surface at the support contact regions; subsequently, all degrees of freedom (DOFs) of the reference point were constrained. A uniform internal pressure of 1.0 MPa was applied to the inner surface. The structure was discretized using linear quadrilateral shell elements (S4R) with a global element size of 50 mm, resulting in a total of 22,917 elements.

### 3.3. Results and Analysis of Static Strength

**Figure 7:**
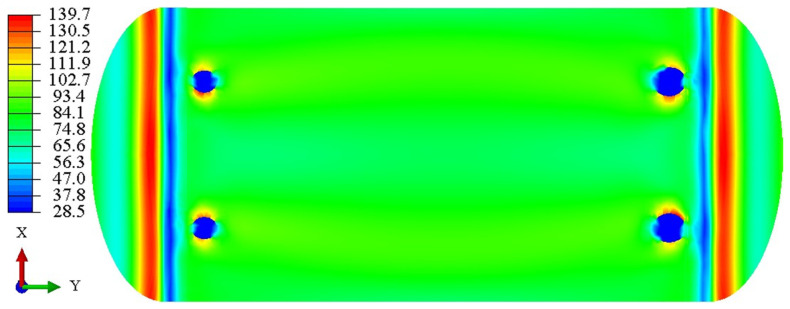
Mises stress cloud diagram on the surface of the LNG storage tank (MPa).

Figure 7 illustrates the Mises stress distribution on the surface of the LNG storage tank. As observed, the peak Mises stress is concentrated within the knuckle region (arc transition) of the ellipsoidal head. If an initial crack initiates at this location, cyclic loading will drive its propagation in two primary directions: through the wall thickness and along the inner surface of the tank.

## 4. Fatigue Crack Growth Simulation in LNG Tanks with Initial Cracks

### 4.1. Finite Element Model of LNG Tanks with Cracks

**Figure 8:**
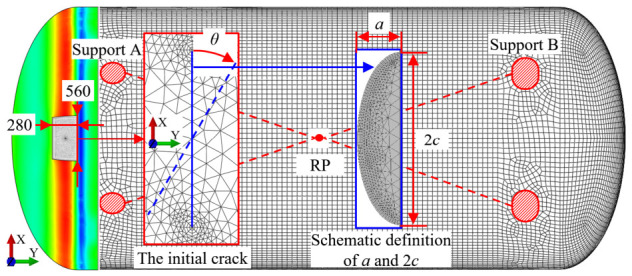
Finite element model of LNG storage tank with an initial semi-elliptical surface crack, local submodel, and schematic definition of crack parameters.

Based on the static strength analysis, the peak Mises stress is identified in the knuckle region—the transition between the ellipsoidal head and the cylindrical shell. Given this stress concentration, this critical site is considered the most vulnerable to failure; thus, an initial crack is introduced here. Figure 8 illustrates the finite element model of the LNG tank featuring the initial crack, including the local submodel, crack dimensions, crack orientation, and schematic definition of crack parameters.

As shown in Figure 8, an initial semi-elliptical surface crack is introduced on the inner wall and oriented normal to the tank surface. The crack orientation, denoted by the angle θ, is defined relative to the positive *x*-axis, which aligns with the major axis of the ellipsoidal head. The crack geometry is characterized by its depth a, representing the maximum penetration from the inner surface, and its surface length 2c, corresponding to the total span of the crack along the inner wall. The gray semi-elliptical inset in Figure 8 is used only as a schematic definition of the crack depth a and surface length 2c, rather than an additional crack or an independent submodel.

The simulation adopts a submodeling technique for local crack-front modeling and finite element remeshing. The submodel region, measuring 280 mm × 560 mm, is placed around the initial crack and discretized with three-dimensional solid elements, while the remaining global tank model retains the shell-element discretization used in the static strength analysis. At the global–submodel interface, shell-to-solid coupling constraints are implemented to ensure kinematic consistency. All other modeling parameters remain identical to those used in the initial static strength analysis.

The local crack-front mesh follows the finite element partitioning strategy reported in [32]. A spider-web mesh is generated around the crack front to resolve the near-front stress and deformation fields required for fracture-parameter evaluation. In this mesh, the innermost layer adjacent to the crack front consists of wedge elements, and the outer rings consist of hexahedral elements. The surrounding crack propagation region is discretized using tetrahedral solid elements, and pyramid elements are used as a transition layer between the spider-web mesh and the tetrahedral mesh. The main parameters of the spider-web mesh include the number of radial element layers Nr, the number of circumferential elements Nc, and the radial element length Lr. According to the grid-independence analysis reported in [32], stable CTOD results can be obtained when Nc and Nr are not smaller than 8 and 3, respectively. Therefore, the same crack-front mesh partitioning principle is adopted in this study to reduce the influence of local mesh density. During crack propagation, the crack geometry is updated incrementally, and the local submodel is remeshed after each crack-growth step.

### 4.2. Stress Intensity Factor Calculation Method

To accurately evaluate the FCG behavior of LNG storage tanks under actual service conditions, the present study considers fatigue loading based on the actual operating environment of the tank. Although wave-induced cyclic stresses are prevalent during transit, relevant literature indicates that low-cycle fatigue governed by loading and unloading cycles is the primary determinant of the service life for such pressure vessels [33]. Consequently, fatigue crack growth simulations in this work were performed under cyclic constant-amplitude loading. Static strength analysis reveals that the stress levels within the tank wall are sufficiently low such that the plastic zone at the crack tip remains negligible relative to the crack dimensions. This justifies the small-scale yielding (SSY) assumption, thereby validating the application of the stress intensity factor (SIF) for analyzing fatigue crack propagation. In this work, the *M*-integral method is implemented to accurately compute the SIF.

The *M*-integral is an interaction integral derived from the *J*-integral, originally proposed by Knowles and Sternberg [34]. A key advantage of the *M*-integral is its path-independence over any closed contour enclosing the crack tip. In this study, the *M*-integral evaluation is coupled with the spider-web crack-front mesh described in Section 4.1. The first element ring adjacent to the crack front is used to represent the near-front field, while the second element ring of the spider-web mesh is selected as the domain-integration region for extracting the stress intensity factors at the crack-front nodes. This treatment avoids direct integration over the most singular element ring and is consistent with the crack-front mesh partitioning strategy reported in [32].

For linear elastic materials, the evaluation of the *M*-integral involves the superposition of a virtual auxiliary field onto the actual state obtained from the finite element analysis. Consequently, the total stress, strain, and displacement fields, as well as the resultant stress intensity factors, are defined as the sum of these two independent mechanical states:

(1) $$\left\{\begin{array}{l} \sigma_{ij}=\sigma_{ij}^{\left(1\right)}+\sigma_{ij}^{\left(2\right)}\\ \varepsilon_{ij}=\varepsilon_{ij}^{\left(1\right)}+\varepsilon_{ij}^{\left(2\right)}\\ u_{i}=u_{i}^{\left(1\right)}+u_{i}^{\left(2\right)}\\ K_{i}=K_{i}^{\left(1\right)}+K_{i}^{\left(2\right)} \end{array}\right.$$

where the superscript (1) denotes the finite element solution, and the superscript (2) denotes the auxiliary field solution; *σ_ij_* and *ε_ij_* are the components of stress and strain, respectively; *u_i_* is the displacement component; *K_i_* represents *K*_I_, *K*_II_, and *K*_III_. Substituting the stress, strain, and displacement components into the J-integral expression yields:

(2) $$\begin{array}{l} J=J^{\left(1\right)}+J^{\left(2\right)}+M^{\left(1,2\right)}\\ J^{\left(1\right)}=\int_{\Gamma }\left(W^{\left(1\right)}n_{1}-T_{i}^{\left(1\right)}\frac{\partial u_{i}^{\left(1\right)}}{\partial x_{1}}\right)ds\\ J^{\left(2\right)}=\int_{\Gamma }\left(W^{\left(2\right)}n_{1}-T_{i}^{\left(2\right)}\frac{\partial u_{i}^{\left(2\right)}}{\partial x_{1}}\right)ds\\ M^{\left(1,2\right)}=\int_{\Gamma }\left(W^{\left(1,2\right)}n_{1}-T_{i}^{\left(1\right)}\frac{\partial u_{i}^{\left(2\right)}}{\partial x_{1}}-T_{i}^{\left(2\right)}\frac{\partial u_{i}^{\left(1\right)}}{\partial x_{1}}\right)ds \end{array}$$

where *J*^(1)^ denotes the J-integral value obtained from the finite element solution; *J*^(2)^ denotes the J-integral value of the auxiliary field J; *M*^(1,2)^ represents the interaction integral between the finite element field and the auxiliary field, which is defined as the *M*-integral; *W*^(1,2)^ is the interaction strain energy density, which can be calculated from the following equation:

(3) $$W^{\left(1,2\right)}=\sigma_{ij}^{\left(1\right)}\varepsilon_{ij}^{\left(2\right)}=\sigma_{ij}^{\left(2\right)}\varepsilon_{ij}^{\left(1\right)}$$

By introducing the *q*-function, the path-integral forms of the above *M*-integral and J-integral are transformed into volume domain integrals:

(4) $$\begin{array}{c} J^{\left(k\right)}=\int_{V}\left(\sigma_{ij}^{\left(k\right)}\frac{\partial u_{i}^{\left(k\right)}}{\partial x_{1}}-W^{\left(k\right)}\delta_{1j}\right)\frac{\partial q}{\partial x_{j}}\;dV\;i,j=1,2,3\;\;\;k=1,2\\ M^{\left(1,2\right)}=\int_{V}\left(\sigma_{ij}^{\left(1\right)}\frac{\partial u_{i}^{\left(2\right)}}{\partial x_{1}}+\sigma_{ij}^{\left(2\right)}\frac{\partial u_{i}^{\left(1\right)}}{\partial x_{1}}-W^{\left(1,2\right)}\delta_{1j}\right)\frac{\partial q}{\partial x_{j}}\;dV\;i=1,2,3\;j=1,2\\ A_{q}=\int_{L}q_{t}\;ds \end{array}$$

**Figure 9:**
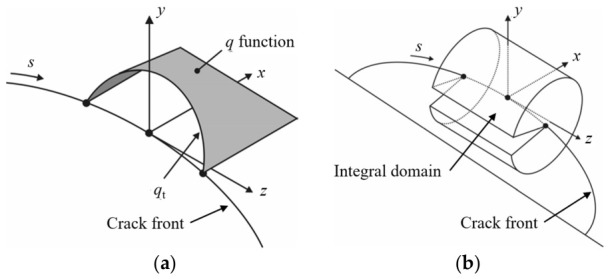
Three-dimensional *M*-integral: (**a**) q-function and (**b**) integration domain.

In the above expressions: *δ_ij_* is the Kronecker delta; *q* is a function, as illustrated in Figure 9a, which can be regarded as a virtual crack extension—equal to 1 at the stress intensity factor evaluation point and 0 at the outer boundary of the integration domain; *q_t_* denotes the value of along the crack front; and *A_q_* represents the area of the virtual crack extension. As shown in Figure 9b, the integration domain for evaluating the three-dimensional *M*-integral is a cylindrical domain enclosing the local crack-front segment. In the present simulations, this domain corresponds to the second element ring of the spider-web mesh around each crack-front node and is updated together with the local remeshed crack-front mesh during crack propagation.

The crack-front mesh density was selected according to the grid-independence analysis reported in [32], in which stable CTOD results were obtained when the number of circumferential elements Nc and the number of radial element layers Nr were not smaller than 8 and 3, respectively. Therefore, no separate mesh-density variation was introduced for each LNG tank case; instead, the same validated crack-front mesh partitioning principle was used throughout all parametric simulations. Since the stress intensity factors are obtained from the path-independent *M*-integral over the second element ring rather than from nodal values in the singular near-front region, this setting reduces the influence of local mesh density on the calculated stress intensity factors.

To obtain *K*_I_^(1)^, *K*_II_^(1)^, and *K*_III_^(1)^, the solution of the auxiliary field (2) is further decomposed into three parts: (2a)–(2c):

(5) $$\begin{array}{lll} K_{I}^{\left(2a\right)}=1, & K_{II}^{\left(2a\right)}=0, & K_{III}^{\left(2a\right)}=0\\ K_{I}^{\left(2b\right)}=0, & K_{II}^{\left(2b\right)}=1, & K_{III}^{\left(2b\right)}=0\\ K_{I}^{\left(2c\right)}=0, & K_{II}^{\left(2c\right)}=0, & K_{III}^{\left(2c\right)}=1 \end{array}$$

Under the assumption of small-scale yielding, the J-integral can be expressed in terms of the energy release rate:

(6) $$G=J=\frac{1-\mu^{2}}{E}K_{I}^{2}+\frac{1-\mu^{2}}{E}K_{II}^{2}+\frac{1+\mu }{E}K_{III}^{2}$$

Then the *M*-integral can be transformed into an expression for the stress intensity factor:

(7) $$M^{\left(1,2\alpha \right)}=\frac{2\left(1-\mu^{2}\right)}{E}\left[K_{I}^{\left(1\right)}K_{I}^{\left(2\alpha \right)}+K_{II}^{\left(1\right)}K_{II}^{\left(2\alpha \right)}+\frac{K_{III}^{\left(1\right)}K_{III}^{\left(2\alpha \right)}}{1-\mu }\right]\;\alpha =a,b,c$$

For homogenous materials, *M*^(1,2)^ is equal to *M*^(1,2α)^, so the stress intensity factor can be calculated using the following formula:

(8) $$\begin{array}{l} K_{I}=\frac{E}{2\left(1-\mu^{2}\right)}\frac{M^{\left(1,2a\right)}}{A_{q}}\\ K_{II}=\frac{E}{2\left(1-\mu^{2}\right)}\frac{M^{\left(1,2b\right)}}{A_{q}}\\ K_{III}=\frac{E}{2\left(1+\mu \right)}\frac{M^{\left(1,2c\right)}}{A_{q}} \end{array}$$

### 4.3. Method for Calculating the Number of Load Cycles

In this study, the Paris law is employed to calculate the crack growth rate at each node *i* (*i* = 1, 2, …, *n*) along the crack front.

(9) $$\begin{array}{l} \frac{da_{i}}{dN}=C{\left(\Delta K_{i}\right)}^{m}\\ \Delta K_{i}=\left(1-R\right)K_{max,i} \end{array}$$

where d*a_i_*/d*N* denotes the crack growth rate at node *i*; Δ*K_i_* is the stress intensity factor range at node *i*; *R* is the stress ratio; *K*_max,*i*_ is the stress intensity factor at node *i* under maximum load; and *C* and *m* are the Paris law parameters. The number of load cycles can then be calculated from the following equation:

(10) $$dN=\frac{da_{i}}{C{\left(\Delta K_{i}\right)}^{m}}$$

### 4.4. Fatigue Crack Growth Simulation Process

**Figure 10:**
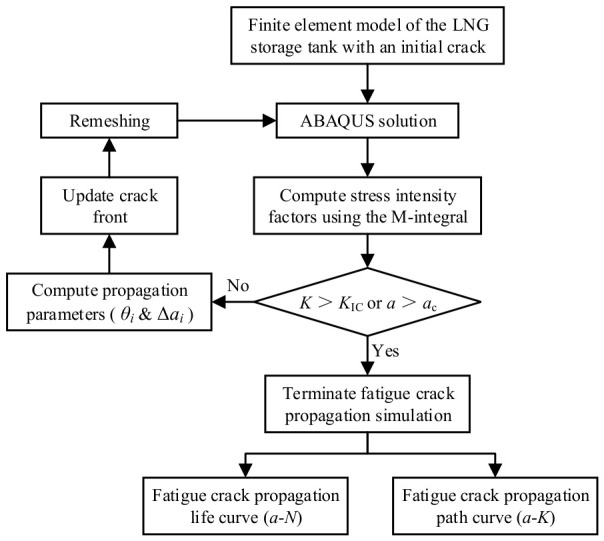
Fatigue crack propagation simulation process for LNG storage tanks with initial cracks.

For the simulation of fatigue crack growth in a cracked LNG storage tank, a corresponding analysis program was developed in this study, and its flowchart is presented in Figure 10. The specific implementation procedure is as follows:

(1) Compute the CTOD at each node *i* (*i* = 1, 2, …, *n*) along the crack front.

(2) Apply the VCTD criterion to determine the crack propagation angle *θ_i_* at each node *i*.

(3) Employ the *M*-integral to calculate the stress intensity factor range Δ*K_i_* at each crack-front node. When the crack extension length Δ*a*_m_ (corresponding to the stress intensity factor range Δ*K*_m_) is specified for a given node, the extension lengths Δ*a_i_* at the remaining crack-front nodes can be computed from Equation (11).

(11) $$\Delta a_{i}={\left(\frac{\Delta K_{i}}{\Delta K_{m}}\right)}^{m}\Delta a_{m}$$

(4) Determine the updated coordinates of each crack-front node after extension based on *θ_i_* and Δ*a_i_*.

(5) Fit the updated crack-front node coordinates to obtain the extended crack-front curve.

(6) Extrapolate the fitted crack-front curve to the structural surface.

(7) Discretize the extrapolated crack-front curve into an appropriate number of geometric points; these points, together with the original crack surface, form new triangular facets, thereby achieving geometric extension of the crack surface.

(8) Introduce the extended crack-surface geometry into the submodel and perform remeshing.

(9) Merge the submodel with the cracked geometry into the global model to realize crack advancement.

(10) Repeat the crack growth steps until the termination criteria for the fatigue crack growth simulation are satisfied, and then compute the fatigue crack growth life.

### 4.5. Validation of the Crack-Growth Framework

To verify the crack-path prediction capability of the proposed crack propagation framework, a benchmark simulation was conducted using a modified compact tension (CT) specimen with an eccentric circular hole. The modified CT specimen is a commonly used benchmark for mixed-mode crack growth because the circular hole changes the local stress field and causes the crack to deflect toward or away from the hole. The specimen geometry and reference crack path were taken from the published modified CT benchmark reported by Baptista et al. [23], in which fatigue crack growth paths were generated by finite element analysis using the VCTD propagation criterion and compared with available experimental and numerical examples in the literature. Therefore, this validation case is used here as a published numerical benchmark for crack-path prediction rather than as a direct independent fatigue test.

**Figure 11:**
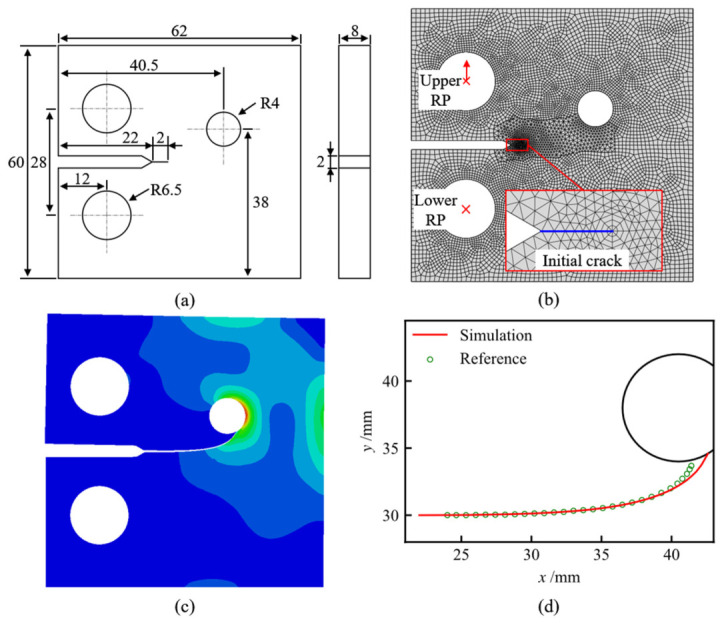
Validation of the crack propagation framework using a modified CT benchmark: (**a**) geometry of the modified CT specimen; (**b**) finite element model of the modified CT specimen; (**c**) stress distribution when the crack approaches the circular hole; and (**d**) comparison between the present simulation and the published reference crack path.

As shown in Figure 11a, the modified CT specimen has a thickness of 8 mm and contains an initial single edge through crack and three circular holes. The circular hole ahead of the crack tip is used to introduce mixed-mode crack propagation. The material was modeled as linear elastic AISI 4340 steel, with an elastic modulus of 207 GPa and a Poisson’s ratio of 0.29. The finite element model is shown in Figure 11b. A concentrated load of 10 kN was applied to the upper reference point and transferred to the loading hole through nodal coupling, while the lower reference point was fixed. The crack-front region was discretized using the same local meshing strategy as that described in Section 4.1 and Section 4.2. The number of radial element layers in the spider-web mesh was set to 3, the number of circumferential elements was set to 8, the radial element length Lr was 0.12 mm, and dtip was 0.16 mm. Since the purpose of this benchmark is to examine the crack-path prediction module, the crack growth process was simplified as quasi-static crack extension, and fatigue cycles were not evaluated in this validation case.

Figure 11c shows the Mises stress distribution when the crack propagates toward the circular hole. A pronounced stress concentration appears around the crack tip and the hole, indicating that the local stress field drives the crack to deflect toward the hole. The predicted crack propagation path is compared with the published reference path in Figure 11d. The present simulation captures both the initial nearly horizontal crack extension and the subsequent curved propagation toward the hole, showing good agreement with the reference benchmark. This result indicates that the VCTD-based direction prediction and the remeshing-based crack geometry update used in the present framework can reproduce the crack path under mixed-mode conditions.

This benchmark mainly verifies the crack-path prediction component of the present framework. The three-dimensional crack-front remeshing and crack-shape update strategy adopted in this work is further supported by the previous validation in [32], where quasi-static tensile tests and corresponding numerical simulations were conducted on cracked stiffened panels. In that study, the simulated load–CMOD response, crack length–CMOD response, and surface crack profiles were compared with experimental measurements, and the simulation results showed good agreement with the test observations. Accordingly, in the following LNG tank simulations, the benchmark-verified crack-path prediction procedure is combined with the experimentally fitted Paris-law parameters in Section 2.2 to calculate fatigue crack growth life.

## 5. Simulation Results and Analysis of Fatigue Crack Growth in LNG Tanks with Initial Cracks

### 5.1. The Influence of the Initial Crack Length on the Fatigue Crack Growth Life

To investigate the influence of the initial surface length on fatigue crack growth life, the initial crack depth was maintained at a constant 1.0 mm. Seven distinct groups of semi-elliptical surface cracks were analyzed, with semi-surface lengths (*c*) of 1.0 mm, 1.25 mm, 2.0 mm, 2.5 mm, 3.0 mm, 4.0 mm, and 5.0 mm. Fatigue crack growth simulations were subsequently executed under a cyclic load ranging from a maximum pressure of 1.0 MPa to a minimum of 0.1 MPa, corresponding to a load ratio (*R*) of 0.1.

**Figure 12:**
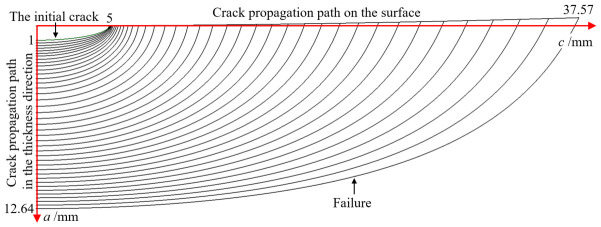
Crack shape changes during fatigue crack propagation simulation when *c*_0_ is 5 mm.

Figure 12 illustrates the evolution of the crack morphology during the fatigue crack growth simulation for the case where *c*_0_ = 5.0 mm. Owing to the symmetry of the model, only one-half of the crack profile is displayed. The figure clearly demonstrates that the crack front undergoes significant shape changes as it propagates. Furthermore, the defined propagation trajectories in both the depth direction and along the tank surface are explicitly indicated in the diagram.

**Figure 13:**
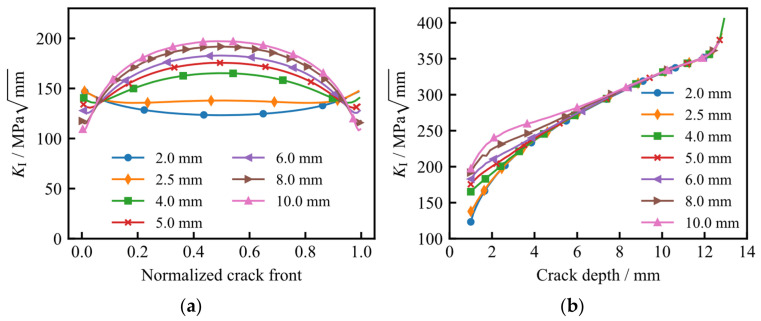
Variation in *K*_I_ for different initial crack surface lengths: (**a**) along the initial crack front; (**b**) along the depth-wise crack-growth path.

Figure 13 illustrates the variation in the Mode I stress intensity factor (*K*_I_) along the initial crack front and the depth-wise crack path for different initial crack surface lengths. In Figure 13a, the normalized crack-front coordinates of 0 and 1 correspond to the tank surface, while a value of 0.5 denotes the deepest point of the crack. As observed, increasing the initial surface length leads to an elevation of *K*_I_ at the deepest point, whereas *K*_I_ at the surface locations decreases. As the crack penetrates further, the *K*_I_ values along the depth-wise path tend to converge. Notably, when the crack depth reaches 12.6 mm, the growth rate of *K*_I_ accelerates significantly (Figure 13b).

**Figure 14:**
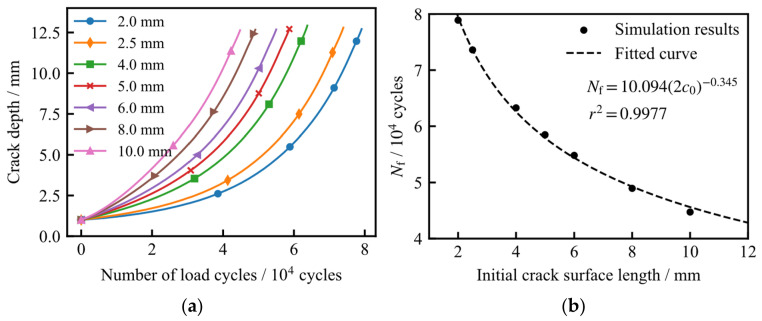
Fatigue crack growth life for different initial crack surface lengths: (**a**) relationship between crack depth and number of load cycles; (**b**) relationship between fatigue crack growth life and initial crack surface length.

Figure 14a illustrates the relationship between crack depth and the number of load cycles (*a*–*N* curves). The results indicate that an increase in the initial surface length accelerates the crack growth rate; consequently, fewer cycles are required to reach a specific depth. In this study, the fatigue crack growth life is defined by the failure criterion of the crack penetrating to a depth of 12.6 mm (representing 90% of the wall thickness). Figure 14b depicts the fatigue life as a function of the initial semi-surface length (*c*_0_). Regression analysis reveals that the fatigue life follows a power-law relationship with respect to *c*_0_.

### 5.2. The Influence of Initial Crack Direction on the Fatigue Crack Growth Life

To evaluate the influence of the initial crack orientation on fatigue life, the initial crack depth and semi-surface length were maintained at 1.0 mm and 4.0 mm, respectively. Five distinct orientations were considered, with initial inclination angles (*θ*_0_) of 0°, 10°, 15°, 20°, and 30°. The fatigue crack growth simulations were executed under a constant-amplitude cyclic load, fluctuating between a maximum pressure of 1.0 MPa and a minimum of 0.1 MPa.

**Figure 15:**
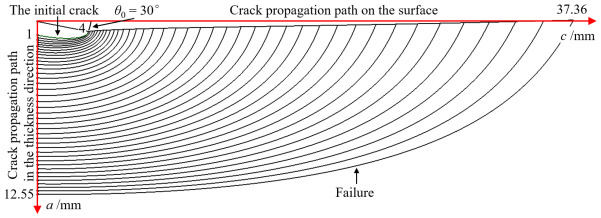
Crack shape changes during crack propagation simulation when *θ*_0_ is 30°.

Figure 15 illustrates the evolution of the crack morphology for the case where *θ*_0_ = 30°; due to symmetry, only one-half of the crack is shown. As observed, when the initial crack is not aligned with the major axis of the ellipsoidal head (i.e., *θ*_0_ > 0°), the crack undergoes a reorientation during propagation. Specifically, the crack deflection angle gradually diminishes, with the crack plane progressively aligning itself toward the 0° direction throughout the growth process.

**Figure 16:**
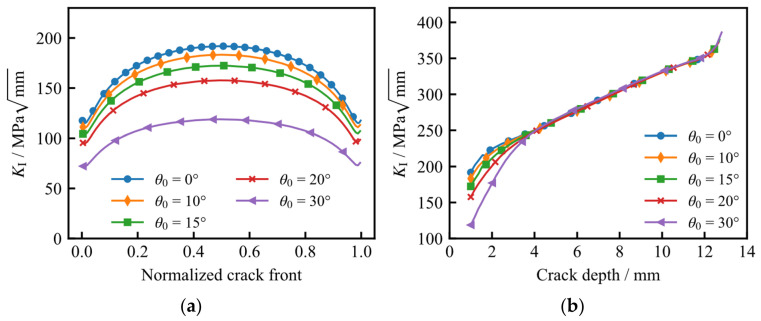
Variation in *K*_I_ for different initial crack directions: (**a**) along the initial crack front; (**b**) along the depth-wise crack path.

Figure 16 illustrates the variation in the Mode I stress intensity factor (*K*_I_) along the initial crack front and the depth-wise crack path for different values of *θ*_0_. As observed, the overall magnitude of *K*_I_ along the crack front decreases monotonically as the initial inclination angle *θ*_0_ increases. However, once the crack propagates beyond a certain depth, the *K*_I_ values along the depth-wise path for different orientations tend to converge and become essentially identical. Notably, when the crack depth reaches 12.6 mm, the growth rate of *K*_I_ accelerates significantly, regardless of the initial orientation.

**Figure 17:**
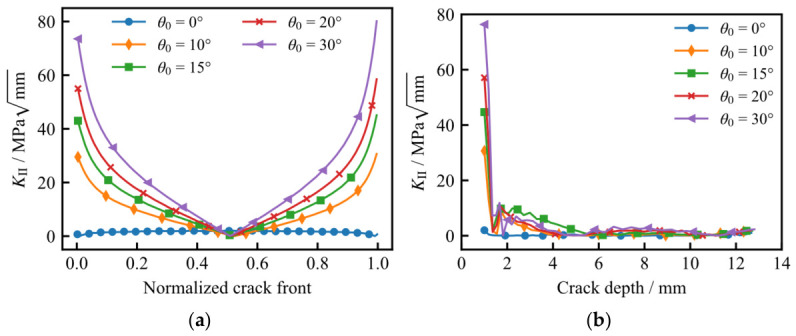
Variation in *K*_II_ for different initial crack directions: (**a**) along the initial crack front; (**b**) along the depth-wise crack path.

Figure 17 illustrates the variation in the Mode II stress intensity factor (*K*_II_) along the initial crack front and the depth-wise crack path for different values of *θ*_0_. As observed, an increase in *θ*_0_ elevates the *K*_II_ magnitude at the tank surface; conversely, *K*_II_ in the depth direction remains negligible and near zero. During the propagation process, the surface *K*_II_ values diminish rapidly, indicating a transition in the crack growth behavior toward a Mode I-dominated regime.

**Figure 18:**
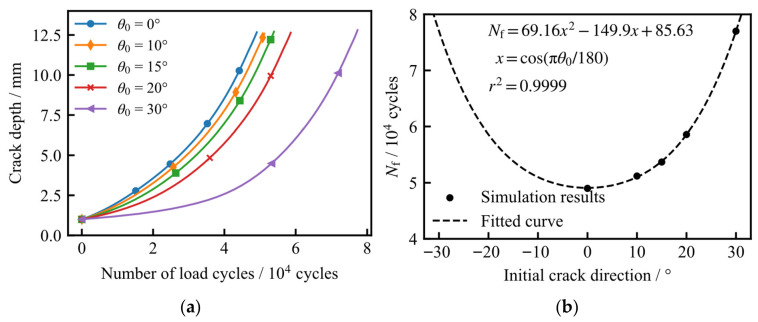
Fatigue crack growth life under different initial crack directions: (**a**) relationship between crack depth and number of load cycles; (**b**) relationship between fatigue crack growth life and initial crack direction.

Figure 18 presents the calculated fatigue crack growth life as a function of *θ*_0_. As observed, an increase in *θ*_0_ leads to a reduction in the average crack growth rate; consequently, the fatigue endurance increases, as more load cycles are required for the crack to reach the target depth. Regression analysis demonstrates that the fatigue life follows a quadratic relationship with cos(*θ*_0_).

### 5.3. The Impact of Maximum Load on Residual Life

**Figure 19:**
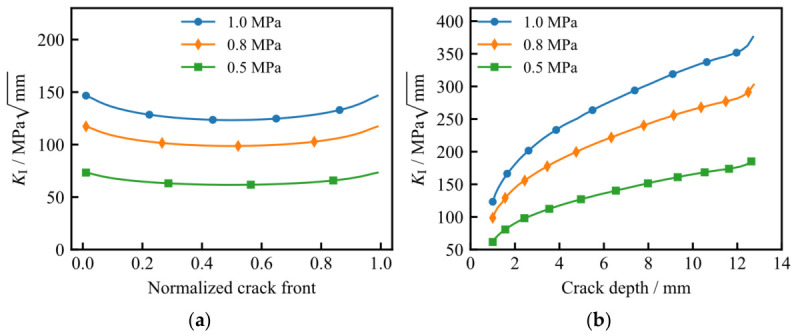
Comparison of *K*_I_ values under different maximum loads: (**a**) variation along the initial crack front; (**b**) variation along the depth-wise crack path.

To assess the influence of the maximum operating load, *P*_max_, on the fatigue life of the LNG storage tank, the initial crack depth and semi-surface length were both fixed at 1.0 mm, with the minimum load *P*_min_ maintained at 0.1 MPa. Three loading scenarios were evaluated, with *P*_max_ values of 0.5, 0.8, and 1.0 MPa. Figure 19 illustrates the distribution of the Mode I stress intensity factor (*K*_I_) along the initial crack front and the depth-wise crack path under these varying loads. As expected, a reduction in *P*_max_ leads to a consistent decrease in *K*_I_ across both the crack front and the propagation trajectory. Notably, as the crack depth reaches 12.6 mm, the *K*_I_ values exhibit a sharp, accelerated increase.

**Figure 20:**
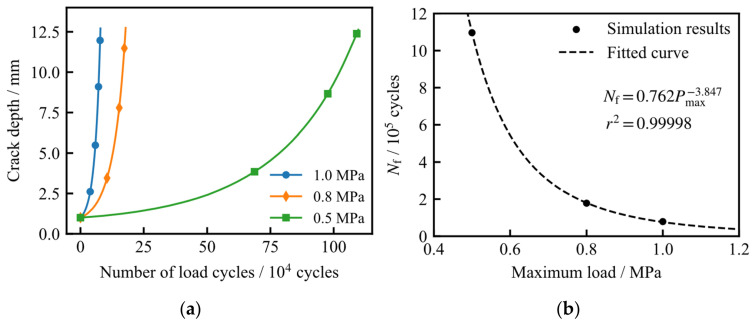
Fatigue crack growth life under different maximum loads: (**a**) relationship between crack depth and number of load cycles; (**b**) relationship between fatigue crack growth life and maximum load.

Figure 20a illustrates the calculated fatigue crack growth life under varying maximum loads (*P*_max_). As observed, a reduction in the maximum load leads to a significant decrease in the crack growth rate; consequently, the fatigue endurance is extended, as more load cycles are required to reach a specific crack depth. As depicted in Figure 20b, the fatigue life (*N*_f_) exhibits a clear power-law relationship with respect to *P*_max_, indicating a strong sensitivity of the tank’s service life to the peak operating pressure.

## 6. Residual Life Prediction Based on XGBoost Regression Model

### 6.1. XGBoost Regression Model

Given a dataset of *n* observations {(**x***_i_*, $y_{i}$)}, where **x***_i_* denotes a feature vector of multiple independent variables and $y_{i}$ is the corresponding dependent variable, the objective of the XGBoost regression model is to construct an approximation function, $\hat{f}(x_{i})$. This function estimates the fitted value, ${\hat{y}}_{i}$, to minimize the discrepancy between the ground truth $y_{i}$ and the prediction ${\hat{y}}_{i}$. In machine learning, the loss function quantifies the error between predicted and actual values; a lower loss signifies superior model fidelity. Consequently, the core of the training process involves an optimization task to minimize this loss function across the training set.

XGBoost, an abbreviation for *Extreme Gradient Boosting*, is an advanced implementation of gradient-boosted decision trees. The algorithm iteratively fits the training data by minimizing a predefined objective function. Specifically, this objective function integrates a regularization term with the loss function to penalize model complexity, thereby effectively mitigating the risk of overfitting and enhancing generalization performance. The objective function is formulated as follows:

(12) $$\mathrm{obj}=\sum_{i=1}^{n}L\left(y_{i},{\hat{y}}_{i}\right)+\gamma T+\frac{1}{2}\lambda \sum_{j=1}^{T}w_{j}^{2}$$

where *L* is the loss function, used to measure the difference between the predicted value ${\hat{y}}_{i}$ and the true value $y_{i}$; *T* is the number of leaf nodes in the decision tree; *w_j_* is the weight coefficient of the *j*_th_ leaf node; *γ* and *λ* are regularization parameters that control the complexity of the decision tree and the smoothness of the weight coefficients, respectively.

XGBoost employs an additive training strategy, where a new decision tree is integrated at each iteration to minimize the objective function—which consists of the cumulative predictions from existing trees plus a regularization term. To achieve faster and more precise optimization, a second-order Taylor expansion is utilized to approximate the loss function. For a differentiable loss function, *L*, its second-order Taylor expansion at the current iteration can be formulated as:

(13) $$L\left(y,\hat{y}+\Delta \hat{y}\right)\approx L\left(y,\hat{y}\right)+\frac{\partial L\left(y,\hat{y}\right)}{\partial \hat{y}}\Delta \hat{y}+\frac{1}{2}\frac{\partial^{2}L\left(y,\hat{y}\right)}{\partial {\hat{y}}^{2}}{\left(\Delta \hat{y}\right)}^{2}$$

Given the second-order Taylor expansion of the loss function ${\hat{y}}^{\left(k-1\right)}$, then the objective function at the *k*_th_ iteration is:

(14) $$\begin{array}{l} {\mathrm{obj}}^{\left(k\right)}=\sum_{i=1}^{n}L\left(y_{i},{\hat{y}}_{i}^{\left(k-1\right)}+f_{k}\left(x_{i}\right)\right)+\gamma T+\frac{1}{2}\lambda \sum_{j=1}^{T}w_{j}^{2}\\ {\mathrm{obj}}^{\left(k\right)}\approx \sum_{i=1}^{n}\left[g_{i}f_{k}\left(x_{i}\right)+\frac{1}{2}h_{i}f_{k}^{2}\left(x_{i}\right)\right]+\gamma T+\frac{1}{2}\lambda \sum_{j=1}^{T}w_{j}^{2} \end{array}$$

Here, *g_i_* and *h_i_* denote the first- and second-order derivatives (gradient and Hessian) of the loss function with respect to the prediction at the previous iteration, respectively. The term $f_{k}(x_{i})$. represents the prediction of the newly added decision tree for the *i*_th_ data point at the *k*_th_ iteration. The final ensemble model computes the sum of predictions from all *K* individual decision trees to finalize the regression training, yielding the consolidated prediction function:

(15) $${\hat{y}}_{i}=\sum_{t=1}^{k}f_{t}\left(x_{i}\right)$$

**Figure 21:**
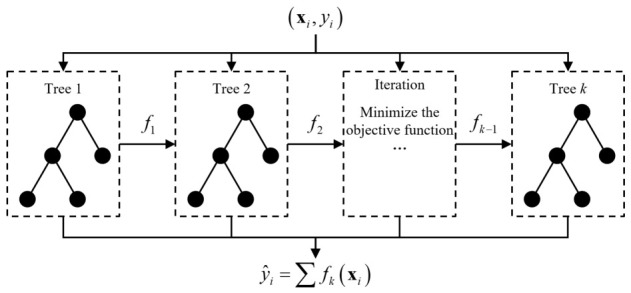
XGBoost algorithm process.

In the formula, *k* represents the number of decision trees included in the model. The process of the XGBoost regression model is illustrated in Figure 21.

### 6.2. Simulation Data Preprocessing

**Figure 22:**
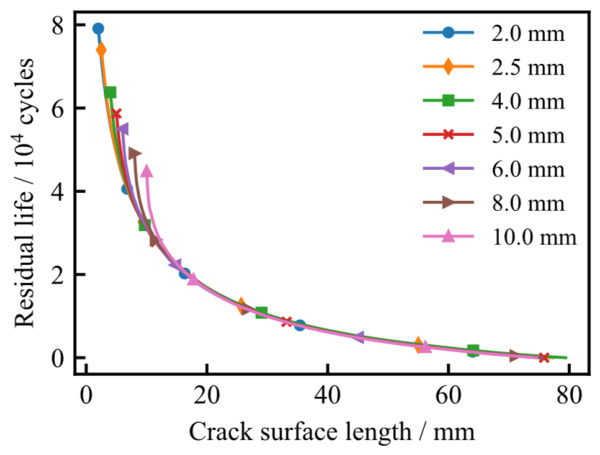
Relationship between residual life and crack surface length under different initial crack surface lengths.

To train the XGBoost regression model, the fatigue crack growth simulation data for various initial surface lengths were processed and structured. The residual life is defined as the number of remaining load cycles required for a crack to grow from its current state to the failure threshold of 12.6 mm. Figure 22 illustrates the relationship between residual life and the instantaneous crack surface length. As observed, as the crack length increases, the residual life curves for different initial configurations tend to converge toward a unified value, indicating that the influence of the initial crack geometry diminishes during the later stages of propagation.

**Figure 23:**
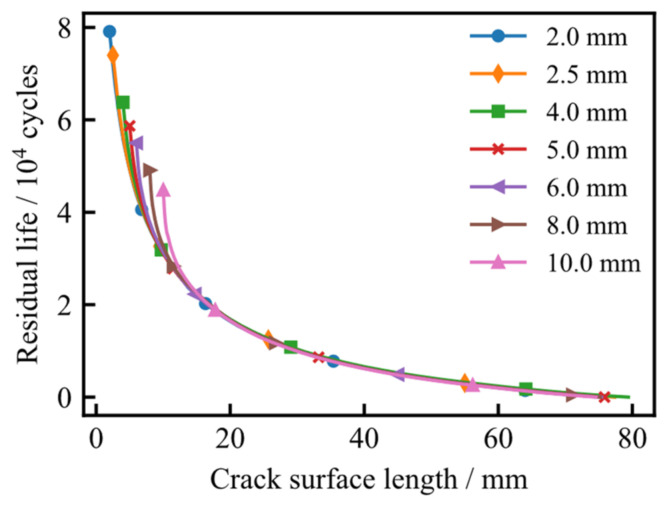
Relationship between residual life and crack depth under different initial crack surface lengths.

Figure 23 illustrates the relationship between residual life and crack depth for various initial surface lengths. As observed, once the crack reaches a specific depth, the residual life trajectories for different initial configurations converge and become essentially identical. This indicates that the residual fatigue life becomes insensitive to the initial crack surface length as the crack approaches the critical penetration stage.

**Figure 24:**
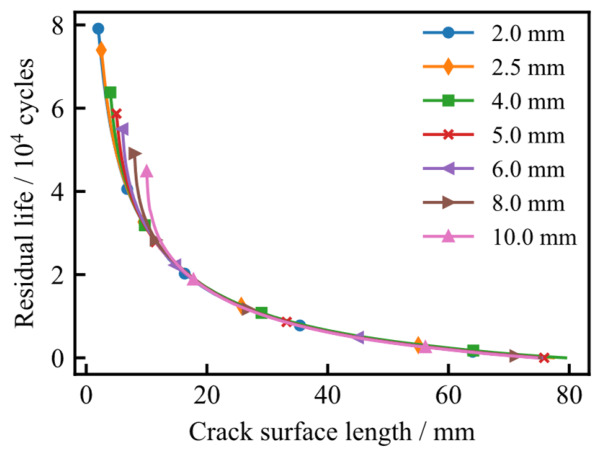
The relationship between crack surface length and depth under different initial crack surface lengths.

Figure 24 illustrates the evolution of the crack surface length relative to the crack depth for various initial configurations. As observed, the fatigue cracks tend to propagate along a preferential and stable trajectory. This indicates that regardless of the initial dimensions, the crack aspect ratio evolves toward a stable, self-similar shape during the growth process.

By establishing the functional correlations between the crack surface length (*c*) and both the residual life (*N*_f_) and crack depth (*a*), a robust and efficient predictive framework is proposed for the structural health monitoring of LNG storage tanks. This approach enables the estimation of the residual service life based on the initial crack state and its observable surface length during propagation, while simultaneously predicting the sub-surface growth in the depth direction. Utilizing the XGBoost regression algorithm, a dual-target predictive model was developed; the specific mathematical expressions for residual life and crack depth are formulated in Equations (16) and (17), respectively.

(16) $$N_{f}=F_{N}\left(2c_{0},2c\right)$$

(17) $$a=F_{a}\left(2c_{0},2c,N_{f}\right)$$

### 6.3. Model Training

The simulation results from five groups of fatigue crack growth tests—with initial semi-surface lengths (*c*_0_) of 1.0, 2.0, 3.0, 4.0, and 5.0 mm—were partitioned as the training set. The training process for the XGBoost regression model involves parameter optimization by minimizing a predefined objective function. In this study, the Mean Squared Error (MSE) was adopted as the loss component within the objective function, formulated as follows:

(18) $$L\left(y,\hat{y}\right)=\sum_{i=1}^{n}{\left(y_{i}-{\hat{y}}_{i}\right)}^{2}$$

The simulation results for the cases with *c*_0_ = 1.25 mm and *c*_0_ = 2.5 mm were reserved as the test set to evaluate the model’s predictive performance. To prevent overfitting, the learning rate was set to 0.01, and a stochastic subsampling strategy was implemented, where 80% of the training data were randomly selected for each iteration. The Mean Absolute Error (MAE) was monitored throughout the process as a convergence criterion; training was terminated via an early stopping mechanism when the MAE on the validation set ceased to show further improvement. The MAE is defined as follows:

(19) $$\mathrm{MAE}=\frac{1}{n}\sum_{i=1}^{n}\left|y_{i}-{\hat{y}}_{i}\right|$$

The residual life prediction model converged after 1045 iterations with a maximum tree depth of 7, achieving a coefficient of determination (*R*^2^) of 0.9964 on the training set. Similarly, the crack depth prediction model underwent 1231 iterations with a maximum depth of 6, attaining an *R*^2^ value of 0.9995. These high regression coefficients indicate an exceptional fit between the models and the training data.

### 6.4. Prediction Performance

The prediction performance of the trained XGBoost models was evaluated using two test cases with *c*_0_ = 1.25 mm and *c*_0_ = 2.5 mm. These two cases were not included in the training set. In this section, the finite element simulation data are consistently referred to as simulated results, while the outputs of the XGBoost models are referred to as predicted results.

**Figure 25:**
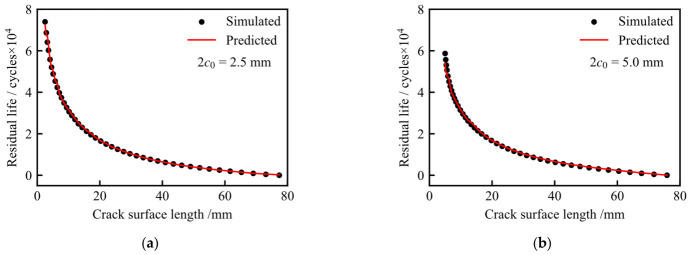
Prediction performance of the residual life model as a function of crack surface length: (**a**) *c*_0_ = 1.25 mm; (**b**) *c*_0_ = 2.5 mm.

Figure 25 illustrates the prediction performance of the residual life model as a function of crack surface length. As the crack surface length increases, the residual life decreases rapidly at the early stage and then gradually approaches zero near the failure threshold. The predicted results are in good agreement with the simulated results for both test cases, indicating that the XGBoost model can accurately reproduce the nonlinear relationship between crack surface length and residual life.

**Figure 26:**
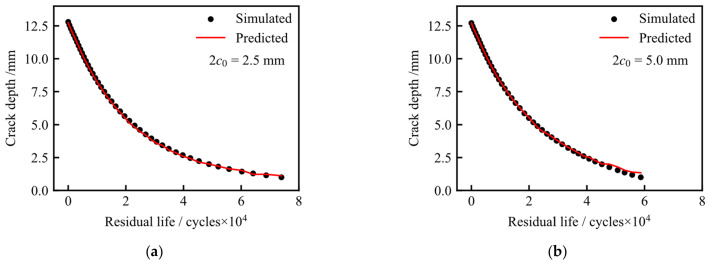
Prediction performance of the crack depth model as a function of residual life: (**a**) *c*_0_ = 1.25 mm; (**b**) *c*_0_ = 2.5 mm.

Figure 26 illustrates the prediction performance of the crack depth model as a function of residual life. In this model, crack depth is the predicted variable and is plotted on the *y*-axis, while residual life is used as the horizontal coordinate to describe the remaining number of cycles before reaching the failure threshold. Since a larger residual life corresponds to an earlier crack-growth stage, the crack depth decreases as the residual life increases. The predicted results agree well with the simulated results, demonstrating that the XGBoost model can effectively estimate crack depth during fatigue crack growth.

## 7. Conclusions

Leveraging a self-developed 3D crack growth program, this study established a comprehensive fatigue simulation procedure for LNG storage tanks. The influence of initial geometry and loading conditions on fatigue endurance was systematically evaluated, culminating in the development of a machine-learning-based life prediction framework. The primary findings are summarized as follows:

(1) Under design conditions, the peak stress in the LNG tank is localized within the arc transition region of the ellipsoidal head. Crack propagation exhibits a marked directional bias, with the surface growth rate significantly exceeding that in the depth direction.

(2) The fatigue crack growth life is sensitive to initial defect configurations and load magnitudes. Specifically, the fatigue crack growth life follows a power-law relationship with both the initial surface length and the maximum load. In addition, the fatigue crack growth life is quadratically correlated with the cosine of the initial crack direction angle, cos*θ*_0_, with larger angles suppressing the depth-wise growth rate.

(3) For semi-elliptical surface cracks under identical loads, the Mode I stress intensity factor *K_I_* along the depth-wise path tends to converge to a unified value after the crack propagates to a certain depth, regardless of the initial crack surface length or initial crack direction. A sharp acceleration in *K_I_* is observed when the crack depth reaches 12.6 mm, corresponding to 90% of the wall thickness.

(4) The established XGBoost regression models provide rapid prediction of both residual life and crack depth. By using the initial crack surface length and the current crack surface length as input parameters, the remaining service life before critical penetration can be estimated efficiently.

The present framework is developed under several assumptions that define its scope of applicability. The fatigue crack growth simulations are based on linear elastic fracture mechanics and the small-scale yielding assumption, and the cyclic loading is simplified to constant-amplitude internal pressure loading. The crack configuration is also limited to a single semi-elliptical surface crack introduced at the critical region identified by static strength analysis. In addition, the XGBoost models are trained using simulation data within the parameter ranges considered in this study, and their extrapolation ability outside these ranges remains limited. Future work should consider variable-amplitude loading, multiple or interacting cracks, elastoplastic crack-front behavior, and further validation using full-scale or component-level LNG tank experiments. The prediction model can also be extended by incorporating additional structural, material, and loading parameters to improve its generalization capability for practical residual life assessment.

## Figures and Tables

**Table 1:** Mechanical properties of high manganese austenitic steel.

Property	Symbol	Value	Unit
Elastic Modulus	*E*	179	GPa
Poisson’s Ratio	*μ*	0.3	—
Yield Strength	*σ*_y_	480	MPa
Ultimate Tensile Strength	*σ*_u_	900	MPa
Reduction in Area	*Z*	60	%

## Data Availability

The original contributions presented in this study are included in the article. Further inquiries can be directed to the corresponding author.

## References

[1] Paris P., Erdogan F. (1963). A critical analysis of crack propagation laws. J. Basic Eng..

[2] Forman R.G., Kearney V.E., Engle R.M. (1967). Numerical analysis of crack propagation in cyclic-loaded structures. J. Basic Eng..

[3] Erdogan F., Sih G.C. (1963). On the crack extension in plates under plane loading and transverse shear. J. Basic Eng..

[4] Sih G.C. (1973). Some basic problems in fracture mechanics and new concepts. Eng. Fract. Mech..

[5] Sih G.C. (1974). Strain-energy-density factor applied to mixed mode crack problems. Int. J. Fract..

[6] Li C. (1989). Vector CTD criterion applied to mixed mode fatigue crack growth. Fatigue Fract. Eng. Mater. Struct..

[7] Kawabata T., Tagawa T., Kayamori Y., Ohata M., Yamashita Y., Kinefuchi M., Yoshinari H., Aihara S., Minami F., Mimura H. (2017). Applicability of new CTOD calculation formula to various a0/W conditions and B× B configuration. Eng. Fract. Mech..

[8] Zhen Y., Tian H., Yi H., Cao Y., Zhang S. (2018). Constraint-corrected fracture failure criterion based on CTOD/CTOA. Int. J. Fract..

[9] Silvestre M.N., Hertelé S., Sarzosa D.F. (2021). On the experimental estimation of CTOD fracture parameter using SE (T) specimens based upon only one clip gauge measurement. Eng. Fract. Mech..

[10] Gao J., Ju X., Zuo Z., Zhao X., Duan M. (2022). Experimental investigation on the low temperature fracture performance of Q690 steel for application to long-span high-speed railway bridges in Tibet harsh environment. Structures.

[11] Dover W.D. (1973). Fatigue crack growth under COD cycling. Eng. Fract. Mech..

[12] Vasco-Olmo J.M., Díaz F.A., Antunes F.V., James M.N. (2019). Characterisation of fatigue crack growth using digital image correlation measurements of plastic CTOD. Theor. Appl. Fract. Mech..

[13] Gesell S., Ganesh R., Kuna M., Fedelich B., Kiefer B. (2023). Numerical calculation of ΔCTOD to simulate fatigue crack growth under large scale viscoplastic deformations. Eng. Fract. Mech..

[14] Escalero M., Muniz-Calvente M., Zabala H., Urresti I., Branco R., Antunes F.V. (2021). A methodology for simulating plasticity induced crack closure and crack shape evolution based on elastic–plastic fracture parameters. Eng. Fract. Mech..

[15] Floros D., Ekberg A., Larsson F. (2019). Evaluation of crack growth direction criteria on mixed-mode fatigue crack growth experiments. Int. J. Fatigue.

[16] Malikova L., Veselý V., Seitl S. (2016). Crack propagation direction in a mixed mode geometry estimated via multi-parameter fracture criteria. Int. J. Fatigue.

[17] Ganesh R., Dude D.P., Kuna M., Kiefer B. (2023). ProCrackPlast: A finite element tool to simulate 3D fatigue crack growth under large plastic deformations. Int. J. Fatigue.

[18] Ammendolea D., Greco F., Leonetti L., Lonetti P., Pascuzzo A. (2023). Fatigue crack growth simulation using the moving mesh technique. Fatigue Fract. Eng. Mater. Struct..

[19] Baktheer A., Martínez-Pañeda E., Aldakheel F. (2024). Phase field cohesive zone modeling for fatigue crack propagation in quasi-brittle materials. Comput. Methods Appl. Mech. Eng..

[20] Ince R. (2004). Prediction of fracture parameters of concrete by artificial neural networks. Eng. Fract. Mech..

[21] Gope D., Gope P.C., Thakur A., Yadav A. (2015). Application of artificial neural network for predicting crack growth direction in multiple cracks geometry. Appl. Soft Comput..

[22] Wang B., Xie L., Song J., Zhao B., Li C., Zhao Z. (2021). Curved fatigue crack growth prediction under variable amplitude loading by artificial neural network. Int. J. Fatigue.

[23] Baptista R., Moita P., Infante V. (2023). Fatigue crack growth on modified CT specimens using artificial neural networks. Int. J. Fatigue.

[24] Gan L., Wu H., Zhong Z. (2022). Fatigue life prediction considering mean stress effect based on random forests and kernel extreme learning machine. Int. J. Fatigue.

[25] Liao W., Long X., Jiang C. (2025). A physics-informed neural network method for identifying parameters and predicting remaining life of fatigue crack growth. Int. J. Fatigue.

[26] Dong Y., Liu R., Li Q. (2026). Prediction of residual life and critical crack length using the forward/inverse machine learning based on the configurational force fatigue model. Int. J. Fatigue.

[27] Yahia A.K., Shahjalal M. (2025). Recent developments and challenges in fracture mechanics–based fatigue life prediction. ASRC Procedia Glob. Perspect. Sci. Scholarsh..

[28] (2010). Tensile Test of Metallic Materials: Part 1: Test Method at Room Temperature.

[29] (2009). Metallic Materials—Tensile Testing—Part 1: Method of Test at Room Temperature.

[30] (2017). Metallic Materials Fatigue Testing Fatigue Crack Growth Method.

[31] (2012). Metallic Materials—Fatigue Testing—Fatigue Crack Growth Method.

[32] Liu Y., Li Z., Yi X., Zhou H., Liu J., Zhou W. (2026). Study on three-dimensional crack propagation simulation based on remeshing. Meccanica.

[33] Kim J., Park K.S., Cha I., Choung J. (2025). Structural Integrity Assessments of an IMO Type C LCO_2_ Cargo Tank. J. Mar. Sci. Eng..

[34] Knowles J.K., Sternberg E. (1972). On a class of conservation laws in linearized and finite elastostatics. Arch. Ration. Mech. Anal..

